# Anxiety and depression in adults with cystic fibrosis: a comparison between patients and the general population in Sweden and three other European countries

**DOI:** 10.1186/s12890-015-0117-9

**Published:** 2015-10-14

**Authors:** Lena Backström-Eriksson, Kimmo Sorjonen, Agneta Bergsten-Brucefors, Lena Hjelte, Bo Melin

**Affiliations:** Department of Clinical Neuroscience, Division of Psychology, Karolinska Institutet, Nobels väg 9, S-171 65 Solna, Sweden; Karolinska University Hospital, Stockholm CF-center, Stockholm, S-141 86 Sweden; Department of Clinical Science, Intervention and Technology, Division of Pediatrics, Karolinska Institutet, S-141 86 Stockholm, Sweden

**Keywords:** Cystic fibrosis, CF, Anxiety, Depression, HADS, Psychiatry, Psychology

## Abstract

**Background:**

Cystic fibrosis (CF) is the most common autosomal recessive life-shortening disease among Caucasians. Studies exploring the prevalence of anxiety and depression in adult CF patients are few, show inconsistent findings and rarely include comparisons with general populations. Prevalence and degree of anxiety and depression were investigated in adult CF patients in Sweden, Belgium, Germany and the UK, and compared to corresponding general population data.

**Methods:**

Adult non-transplanted CF patients from the three largest CF-centres (out of four) in Sweden (N = 129; Age range 18–70 years; 50 % women) completed the Hospital Anxiety and Depression Scale (HADS). Studies using HADS in adult CF populations in the UK, Germany, and Belgium were included, as well as HADS normative data from the corresponding general populations.

**Results:**

No elevated risk for anxiety and depression was found among the CF patients. However, a Country x Group interaction effect emerged; CF patients experienced a higher degree of anxiety than the general population in Sweden, but not in the other countries, though this finding did not remain significant in a logistic regression analysis. In Sweden the effect was limited to women. A Country x Group interaction effect was also found for Depression; CF patients experienced lower degree of depression than the general population in Sweden, Germany and the UK, but not in Belgium/Netherlands.

**Conclusions:**

Contrary to earlier outcomes, the present results do not indicate any general elevated risk for anxiety and depression among CF patients. Anxiety was slightly higher in the Swedish CF population, compared to the general population; this finding was not seen in the other countries. Depression among CF patients was lower than or similar to that in the general populations in the studied countries.

## Background

Cystic fibrosis (CF) is the most common autosomal recessive, life-shortening disease among Caucasians [1]. The known genetic defect causes inappropriately thick mucus and malfunctioning of epithelial organs such as the lungs, pancreas and liver. CF is chronic and the expected course of the disease is a progressive deterioration of health resulting in decreasing lung function and capacity. The primary cause of mortality and morbidity in CF is progressive lung disease [2]. CF is still incurable; the treatment offered is symptomatic and aims to postpone disease progression. The treatment is complex, must occur daily and is extremely time consuming and demanding for the patient [3]. However, centralized care and advances in management and treatment of CF have led to a substantial improvement in prognosis and survival during recent decades and, as a result, a growing adult patient population [2].

Chronic respiratory patients are generally at increased risk for anxiety and depression [4–6]. Studies exploring prevalence and levels of anxiety and depression in adult CF patients are few, show inconsistent findings and rarely include comparisons with general populations. According to two reviews, anxiety [7] and depression [7, 8] appear to be more common in CF patients than in the general population, and a recent international multicentre study [9] revealed high rates of depression and anxiety in CF patients. In contrast, a Belgian single-centre study [10] and a recent large UK study [11] showed anxiety and depression rates in CF patients similar to those in the respective general populations. Furthermore, two smaller single-centre studies conducted in Sweden [12] and the US [13] found relatively healthy psychological functioning in CF patients.

Concerning prevalence estimates, two multicentre studies [11, 14] conducted in the UK among 1779 adults with CF and among 670 German CF patients (aged 12–64 years) reported an anxiety prevalence of 34 and 20.6 %, respectively, and depression prevalence of 13 and 9.6 %, respectively. The multicentre study [9] mentioned above found an anxiety prevalence of 32 % and depression prevalence of 19 % among adult CF patients across countries. Two single-centre studies among 57 adult CF patients in Belgium [10] and 121 in the UK [15] reported elevated anxiety levels in 30 and 33 % of the patients, respectively, and depression symptoms in 13 and 16 %, respectively. An earlier review [7] presented prevalence rates for depression ranging from 29 to 46 % and for anxiety from 0 to 31 %. Furthermore, a small single-centre study [16] conducted in the US reported a depression prevalence of 30 %.

In Sweden, only one study has looked at psychosocial issues among CF patients [12]. An evaluation of anxiety and depression rates in the Swedish adult CF population is therefore needed. The aim of the present study, which is in part the Swedish arm of The International Depression/anxiety Epidemiological Study (TIDES) [9], was to compare data on prevalence and degree of anxiety and depression among Swedish CF patients with corresponding Swedish general population data. An additional aim was to include previous anxiety and depression prevalence data from adult CF populations in other countries and corresponding general populations in the comparisons. Due to previous conflicting outcomes and methodological differences, between-population comparisons were performed using similar measurements.

## Method

### Subjects

The three largest CF centres (out of four) in Sweden recruited participants to the study; these were the adult patients in the Swedish arm of TIDES. Patients of interest were adults (≥18 years of age) with a confirmed CF diagnosis. Transplanted patients were excluded because a transplant changes the symptomatology, health status and treatment regimen dramatically. After exclusion, 249 subjects remained as potential participants. Participants were 129 voluntary CF patients: 50 % women; mean age: 30.4 (SD 11.73, range 18–70 years). Mean Forced Expiratory Volume in one second (FEV_1_) per cent of predicted value was 73.3 (SD 27.3, range 22–125).

Non-participant data were available only for the largest of the participating sites, where 27 % of potential patients did not participate; 44 % women; mean age: 29.7 (range 19–65). Of those patients who did not participate, 12 explicitly refused, 3 were living abroad and the reasons the remaining 15 patients did not participate were unknown.

### Procedures

The procedures were in accordance with the TIDES [9] protocol. The patients were invited to participate and informed about the study via regular mail. The CF centre staff then approached them by phone regarding participation. If they were interested in participating, an appointment with the CF team psychologist or social worker was scheduled in connection with a routine outpatient clinic visit. During this appointment and after giving his/her written informed consent/assent, the patient completed the Hospital Anxiety and Depression Scale (HADS) in the presence of a psychologist/social worker who also collected demographic data. Completion of the questionnaire usually took <10 min. Patients who had elevated HADS scores (+8) were followed up by the CF team psychologist. Following data collection, a medical check-up was performed. Data were collected consecutively between March 2008 and February 2009. Ethical approval was obtained from the Central Ethical Review Board, Stockholm (reg. no. 2007/1266–31).

### Measures

Anxiety and depression were measured using the Swedish validated version [17] of HADS [18], a well-established and well-validated screening tool with good psychometric properties [19] developed for use in somatic populations. HADS was developed based on the need for a short, easily administered questionnaire that measures symptoms of emotional distress but excludes symptoms deriving from a physical disorder. The questionnaire consists of two subscales – anxiety and depression – each containing 7 items (range 0–3). Patient results are obtained by summing up each subscale, yielding values from 0–21. Each subscale has three ranges: 0–7 (non-cases), 8–10 (mild-moderate anxiety/depression) and 11–21 (moderate-severe anxiety/depression). The cut-off scores are defined based on the psychiatric ranges of anxiety and depression [18]. HADS scores can be used in two ways: either by comparing an individual’s score to normative values, or by using cut-off scores indicating severity of distress levels [18, 20].

### The previous studies used in the comparisons

The selected comparison countries were chosen because data on them were available in the existing literature; the articles were retrieved by searching PubMed, Medline and Google Scholar. Studies using HADS in adult CF populations were of interest. The next step was to find studies estimating HADS general population scores for the same selected countries. Data on number of subjects, age (range, mean and SD), sex distribution, HADS Anxiety and Depression mean score and prevalence rate were collected from the articles. In two of the studies [14, 21], adolescent data (age <17) were included in the original article; these data were excluded. The Swedish general population data were taken from two different studies [17, 21] to better match the total age range in the present Swedish CF sample. Dutch general population data [22] were used in the comparisons with the Belgium CF sample because the authors of the original article used them as a reference when they concluded that the Belgium CF population scores were similar to those of the general population.

### Statistics

A dataset with the characteristics (*N, M, SD*, and frequencies) presented in Tables 1 and 2 was simulated. Two separate three-way ANOVAs were conducted with Sex, Country, and Group (CF patient/General) as independent variables, and with either HADS anxiety or HADS depression as the dependent variable. Binary logistic regression was used to analyse effects on the odds of having an elevated score (≥8) on anxiety or depression. Effects of age on HADS anxiety and depression, as well as the effects of anxiety and depression on each other, were analysed using ordinary linear regression. All analyses were conducted in IBM SPSS Statistics 22.

**Table 1:** Descriptive statistics for the present Swedish CF population as well as other CF and general populations

		Age		HADS_A		HADS_D	
Population	N	*M*	Range	*M (SD)*	≥ 8 (%)	*M (SD)*	≥ 8 (%)
CF, Sweden (present)	129	30.4	18–70	5.5 (3.7)	27	3.0 (2.9)	9
General, Sweden [17]	624	44.0	30–59	4.6 (3.7)	20	4.0 (3.5)	15
General, Sweden [21]^b^	176	-	20–23	5.5 (4.0)	28.1^a^	3.1 (3.0)	9.5^a^
CF, UK [15]	121	30.0	18–70	6.1 (3.8)	33	3.6 (3.3)	16
CF, UK [11]	1779	-	≥ 18	6.1 (4.1)	34	3.4 (3.3)	13
General, UK [20]	1792	41.5	18–91	6.1 (3.8)	33	3.7 (3.1)	11
CF, Germany [14]^b^	343	-	21–50	4.9 (3.5)	22	3.2 (3.3)	12
General, Germany [23]	4410	50.3	≥ 18	4.7 (3.5)	21	4.7 (3.9)	24
CF, Belgium [10]	57	26.7	-	5.6 (3.9)	30	3.5 (3.6)	13
General, Netherlands [22]	199	39.9	18–65	5.1 (3.6)	24.4^a^	3.4 (3.3)	11.9^a^

^a^Not presented in the article, predicted from the mean value

^b^Excluding the youngest age category

**Table 2:** Descriptive statistics, separate for men and women

	Men			Women		
Population	*N*	HADS-A	HADS-D	N	HADS-A	HADS-D
CF, Sweden (present data)	65	4.5 (3.0)	3.0 (2.8)	64	6.5 (4.1)	3.0 (2.9)
General, Sweden [17]	267	4.3 (3.6)	4.3 (3.6)	357	4.8 (3.8)	3.8 (3.4)
General, Sweden [21]^b^	83^a^	4.6 (3.3)	2.8 (2.4)	93^a^	6.2 (4.5)	3.3 (3.4)
CF, UK [11]	929	5.7 (3.9)	3.4 (3.3)	850	6.6 (4.3)	3.4 (3.4)
General, UK [20]	810	5.7 (3.7)	3.6 (3.2)	978	6.8 (4.1)	4.0 (3.6)
CF, Germany [14]^b^	182	4.6 (3.5)	3.1 (3.3)	161	5.4 (3.5)	3.4 (3.4)
General, Germany [23]	1929	4.4 (3.3)	4.8 (4.0)	2481	5.0 (3.6)	4.7 (3.9)

^a^Not presented in the article, estimated

^b^Excluding the youngest age category

## Results

The three Swedish CF centres recruited a total of 52 % of the potential participants – 72 %, 53 % and 21 % of their clinic populations, respectively.

A dataset with the characteristics (*N, M, SD*, and frequencies) presented in Table 1 was simulated. An ANOVA with mean Anxiety as the dependent variable and with Country, Group (CF patient/General population), and Sex as factors revealed a significant main effect of Sex, *F*(1, 9237) = 49.82, *p* < .001, η^2^ = .005, with women (*M* = 5.66, *SD* = 3.95) experiencing higher levels of anxiety compared with men (*M* = 4.94, *SD* = 3.59). However, the effect of Sex was not moderated by Country or Group (CF patient/General population), and in order to include the Belgian samples, which did not report results separately for men and women, a simplified two-way ANOVA, with Country and Group as factors, was conducted (see Table 2 for descriptive values). This analysis revealed a significant Country × Group interaction effect, *F*(3, 9618) = 2.96, *p* = .031, η^2^ = .001. Separate analyses for each country revealed that CF patients experienced higher levels of anxiety than the general population in Sweden, *F*(1, 927) = 4.56, *p* = .033, η^2^ = .005, but not in the three other countries (all *p*s > .19). When conducting a binary logistic regression separately for the four countries, Group was not found to have a significant effect on the odds of having an elevated score (≥ 8) on Anxiety in any country (all *p*s > .17).

A significant Country × Group interaction effect was found also for Depression, *F*(3, 9618) = 7.45, *p* < .001, η^2^ = .002. Sex had no main effect on Depression and was not involved in any interaction effects with Country or Group. Separate analyses for each country revealed that CF patients experienced lower levels of Depression than the general population in Sweden, *F*(1, 927) = 5.95, *p* = .015, η^2^ = .006, the UK, *F*(1, 3686) = 13.25, *p* < .001, η^2^ = .004, and Germany, *F*(1, 4751) = 45.32, *p* < .001, η^2^ = .009, but not in Belgium, *F*(1, 254) = 0.039, *p* = .844, η^2^ < .001. When conducting a binary logistic regression separately for the four countries, being a CF patient had a significant negative effect on the odds of having an elevated score (≥ 8) on Depression in Germany only, *OR* = 0.450, *p* < .001.

When restricting the analysis to the general populations, Country had a significant effect on anxiety, *F*(3, 7193) = 81.36, *p* < .001, η^2^ = .033, as well as on depression, *F*(3, 7193) = 38.57, *p* < .001, η^2^ = .016. According to Tukey HSD Post Hoc Test, the general UK population had a significantly higher level of anxiety than the three other general populations (*p* < .001, *p* > .5 for the comparisons between Sweden, Germany, and Belgium), while the general German population had a significantly higher level of depression than the three other populations (*p* < .001, *p* > .4 for the other comparisons).

### Sex/Age in the Swedish CF population

When limiting the analyses to the present Swedish CF sample and to data simulating the Swedish general population [17, 21], a Group (CF-patient/general population) × Sex interaction effect on Anxiety was found to be marginally significant, *F*(1, 925) = 3.07, *p* = .080, η^2^ = .003. When analysing the effect of Group separately for women and men, CF patients were found to have a higher mean among women, *F*(1, 512) = 16.17, *p* = .007, η^2^ = .014, but not among men, *F*(1, 413) = 0.18, *p* = .676, η^2^ < .001 (Table 2). A similar analysis with Depression as the dependent variable revealed an effect of Group (see above), but this effect was not moderated by Sex, *F*(1, 925) = 0.10, *p* = .748, η^2^ < .001.

In Table 3, standardized regression coefficients (beta-weights), when predicting HADS-A and HADS-D from each other and age, are presented. Although not always significant, age tended to have a negative association with anxiety and a positive association with depression, while anxiety and depression had a positive association with each other.

**Table 3:** Crude and adjusted effects of age, HADS anxiety, and HADS depression

	Linear^a^		Logistic^b^	
Predictor	HADS-A	HADS-D	HADS-A	HADS-D
Crude				
Age	-.015	.243*	0.795	1.405
HADS-A	-	.499**	-	3.310**
HADS-D	.499**	-	2.127**	-
Adjusted				
Age	-.144^†^	.250**	0.573*	1.543
HADS-A	-	.502**	-	3.417**
HADS-D	.534**	-	2.509**	-

^a^The values are standardized beta-weights

^b^The values stand for the multiplicative change in the odds for anxiety and depression (score ≥ 8) when the predictor increases by one

^†^ *p* < .10; *p < .05; **p < .001

## Discussion

The present findings suggest no elevated risk for anxiety/depression in CF patients compared to their respective general populations, which is contrary to findings from some previous studies [7–9]. However, the results indicate that adult CF patients do have an elevated degree of anxiety compared to the general population in Sweden, although not in the three other countries under study. This outcome should be interpreted with caution, as the effect did not remain when performing the logistic regression analysis, and the significant effect revealed in the ANOVA was very weak. Furthermore, CF patients experienced a lower degree of depression than that found for the normal population in Sweden, the UK and Germany. However, this effect remained in the German sample only when the logistic regression was conducted and might be due to the higher mean age in the German general population, given that depression seems to increase with age [17, 23, 24].

In the Swedish sample, women with CF reported a higher degree of anxiety than did women in the general population, an effect not seen among the men. This is contrary to recent findings among CF patients in the UK [11, 15], where men with CF were more anxious than healthy subjects, and women with CF reported a degree of anxiety similar to normative scores. Furthermore, consistent with previous studies in both CF populations [9, 11, 14, 25] and general populations [17, 23, 24], age tended to be positively associated with depression in the Swedish CF sample. Contrary to other findings among CF patients [9, 11, 14, 15], age tended to be negatively associated with anxiety.

Because the present study did not investigate predictors of anxiety and depression, it is difficult to explain why CF patients showed an elevated degree of anxiety in Sweden but not in the other three countries. From an international perspective, Swedish CF patients have been found to have good pulmonary function, health status and a high survival age [26–28]. The FEV_1_ in the present CF sample was also relatively high and, thus, the elevated degree of anxiety found should not be due to poor health status, but could instead be related to treatment regimen issues. It is well known that CF treatment is demanding [3, 29]. Since the 1980s, the Swedish treatment regimen, on top of the standard treatment, has included frequent physical training as an essential part, and daily airway clearance therapy is based on physical exercise and chest physiotherapy [26]. Furthermore, monthly medical check-ups and home intravenous antibiotic treatment have been standard since the 1980s [30, 31]. It is possible that this expanded treatment burden/responsibility contributes to an elevated degree of anxiety in the patient group. However, this does not explain why elevated anxiety scores were found only among female CF patients in Sweden.

There are some limitations regarding the low levels of depression symptoms found in the present CF populations. First, it is possible that the HADS underestimates depression. In a recent study among CF patients comparing HADS with another screening tool (Patient Health Questionnaire for depression; PHQ-9), discrepancies were found in depression estimates [32]. Furthermore, another recent study [9] revealed that several limitations were found in more recent evaluations of HADS. Second, as already mentioned, some previous studies [17, 23, 24] have indicated that depression increases with age. Another possible explanation for the low depression scores in the present CF samples, as compared to their corresponding general populations, is the higher mean age in some of the present general populations. The depression rates should therefore be interpreted with caution.

This is the first study to systematically investigate the prevalence and degree of anxiety and depression in a Swedish adult CF population. However, at one of the participating sites, both the response rate and depression mean scores were very low, which affected the representativeness negatively. Because refusal data are only available for one centre, selection bias cannot be excluded. The Swedish general population data were taken from two studies with different age ranges so as to better match the total age range in the Swedish CF sample, and our comparisons may therefore have been affected by methodological issues and potential changes in anxiety and depression prevalence over time. Another limitation is the present use of existing HADS normative data instead of matched control subjects, which would have been a preferable approach. The characteristics of the present normative samples differ from those of the corresponding CF populations. For example, the mean ages of the different samples vary and are higher than in some of the present CF populations. The samples also vary in size, and some of the general population data are old compared to the corresponding CF population data.

Because the results do not indicate elevated risks for anxiety and depression among CF patients, it should also be noted that the countries under study are wealthy and provide some kind of public/universal health insurance system, possibly eliminating stressors that may have an impact on psychological wellbeing. Furthermore, previous studies [33–35] have shown great differences across European countries concerning both availability of specialist CF care, and demography, mortality, and health status in the CF populations. Generalizing these results to other CF populations should therefore be done with caution.

From a clinical perspective, anxiety and depression among CF patients should be seen as risk factors that could have a negative impact on adherence to treatment [36–38] and quality of life. Psychological competence should therefore be integrated into CF care. In Sweden, psychologists have been part of CF care for more than two decades, which may have contributed to the low prevalence of anxiety and depression found in the present study. However, more work has to be done because our results do indicate an elevated degree of anxiety in the Swedish CF population.

## Conclusions

On the whole, the present results do not indicate any elevated risks for anxiety and depression within the studied CF populations, and depression mean scores among CF patients were lower than or similar to those of the general population, a finding that requires further research using measurements that are more sensitive to depression symptoms. However, the degree of anxiety was higher among the Swedish CF patients compared to the general population, although this difference was not seen in the UK, Germany and Belgium. Explaining the low prevalence of anxiety and depression found in the present countries requires further research. Such research may help in developing treatment programs for CF populations in which high prevalence of anxiety and depression is a serious problem.

## Abbreviations

ANOVA: Analysis of variance
CF: Cystic fibrosis
HADS: Hospital anxiety and depression scale
